# Intuitive BIM-aided robotic navigation and assets localization with semantic user interfaces

**DOI:** 10.3389/frobt.2025.1548684

**Published:** 2025-03-26

**Authors:** Rafael Gomes Braga, Muhammad Owais Tahir, Sina Karimi, Ulrich Dah-Achinanon, Ivanka Iordanova, David St-Onge

**Affiliations:** ^1^ INIT Robots, Mechanical Engineering Department, École de Technologie Supérieure, Montréal, QC, Canada; ^2^ GRIDD, Construction Engineering Department, École de Technologie Supérieure, Montréal, QC, Canada; ^3^ MISTLab, Computer and Software Engineering Department, Polytechnique Montréal, Montréal, QC, Canada

**Keywords:** semantic navigation, BIM/IFC, path planning, domain knowledge, mobile robots

## Abstract

**Introduction:**

The deployment of mobile robots on construction sites has gained increasing attention from both academic research and industry due to labor shortages and the demand for more efficient project management. However, integrating robotic systems into dynamic and hazardous construction environments remains challenging. Key obstacles include reliance on extensive on-site infrastructure, limited adaptability, and a disconnect between system capabilities and field operators' needs.

**Methods:**

This study introduces a comprehensive, modular robotic platform designed for construction site navigation and asset localization. The system incorporates Building Information Modeling (BIM)-based semantic navigation, active Ultra-Wideband (UWB) beacon tracking for precise equipment detection, and a cascade navigation stack that integrates global BIM layouts with real-time local sensing. Additionally, a user-centric graphical user interface (GUI) was developed to enable intuitive control for non-expert operators, improving field usability.

**Results:**

The platform was validated through real-world deployments and simulations, demonstrating reliable navigation in complex layouts and high localization accuracy. A user study was conducted, confirming improved task efficiency and reduced cognitive load for operators.

**Discussion:**

The results indicate that the proposed system provides a scalable, infrastructure-light solution for construction site robotics. By bridging the gap between advanced robotic technologies and practical deployment, this work contributes to the development of more adaptable and user-friendly robotic solutions for construction environments.

## 1 Introduction

The North American construction industry continues to face significant challenges, including low productivity, labor shortages, safety risks, and substantial environmental impacts (Pradhananga et al., 2021). Despite its vital role in the global economy, the sector has been slow to adopt transformative technologies due to high implementation costs, fragmented processes, and cultural resistance to change. These persistent barriers, coupled with an aging workforce and the physically demanding nature of construction work, exacerbate inefficiencies and limit progress (Pradhananga et al., 2021).

Robotics offers a promising avenue to address these challenges, integrating advanced sensing, navigation, and decision-making capabilities to improve productivity, enhance worker safety, and reduce waste (Kostavelis and Gasteratos, 2017). Robotic systems have already demonstrated significant potential in tasks such as site surveying, tracking Mechanical, Electrical, and Pumbling (MEP) equipment, monitoring worker safety, and transporting materials. However, construction sites are dynamic and unstructured environments, requiring robotic solutions capable of accurate spatial understanding and adaptability to constant changes and complex interactions. The fragmented nature of construction processes further complicates the deployment of robotic systems, necessitating solutions that integrate seamlessly with existing workflows (Pradhananga et al., 2021).

Traditional navigation approaches often rely on geometric maps, which, while useful, lack the semantic richness necessary for handling complex tasks on dynamic construction sites. Building Information Modeling (BIM) and digital twins present a transformative solution by embedding both geometric and semantic data into site representations (Kim and Peavy, 2022; Karimi et al., 2021). These technologies provide robots with domain-specific knowledge of the construction environment, enabling context-aware navigation, informed decision-making, and efficient task execution (Kostavelis and Gasteratos, 2017).

Semantic mapping, which integrates geometric and semantic information, has shown promise in bridging this gap. Modern methods often employ sensors such as cameras and Light Detection And Raging (LiDAR), combined with deep learning techniques (Pu et al., 2023; Zhang and Li, 2023). However, these approaches are computationally demanding and require large datasets, posing challenges for real-time deployment in dynamic environments. BIM-based systems, by contrast, offer a structured and lightweight alternative, allowing robots to directly access pre-existing semantic information (Karimi et al., 2021; Kim and Peavy, 2022). Drawing on the Building Information Robotic System (BIRS) framework (Karimi et al., 2021), this work leverages building lifecycle data to enable robots to perform context-aware operations and enhance site navigation.

In this paper, we propose a comprehensive system for robotic site surveying, combining BIM-based semantic navigation, active equipment detection using Ultra-Wideband (UWB) beacons, and an intuitive teleoperation interface. The system features a cascade navigation stack that integrates global and local path planning, utilizing both topological and metric maps derived from BIM data. Active sensing ensures real-time adaptability by updating maps with newly detected elements during navigation. A semantic graphical user interface (GUI) empowers non-expert users to manage robotic operations and interpret survey results effectively, fostering seamless human-robot collaboration.

The main contributions of this work include:

1. A cascade navigation stack integrating high-level path planning with real-time local adjustments, fully leveraging BIM data, implemented in the Robot Operating System (ROS).
2. An active equipment detection strategy using a minimalist, low-cost UWB-based approach for precise localization of construction assets.
3. A semantic graphical user interface designed to simplify robot operation for non-expert users.

By integrating semantic navigation, active sensing, and user-friendly interfaces, this study addresses key challenges in robotic site surveying and highlights the potential for safer, more efficient, and sustainable construction processes. The paper is organized as follows: Section 2 reviews relevant work; Section 3 details the proposed system and the experimental design; and Section 4 presents the experimental results. Section 6 discusses the findings and outlines directions for future research.

## 2 Related work

### 2.1 Navigating construction sites

Effective navigation in construction settings requires an integrated understanding of spatial and semantic site details. Recent research has proposed diverse strategies to enhance navigation capabilities, with approaches often categorized based on their map structures and organizational frameworks.

Indoor map models, a cornerstone of navigation strategies, are typically classified into grid-based, network-based, and hybrid grid-network-based systems. Grid-based models divide spaces into discrete, non-overlapping cells, offering straightforward representations suitable for pathfinding algorithms. Network-based models, such as the Geometric Network Model (GNM), represent spaces as graphs where nodes correspond to locations and edges encode travel distances (Chen Q. et al., 2022). Hybrid models merge these paradigms to balance spatial accuracy and connectivity. For example, Zhou et al. (2020) proposed a grid-network model that uses the Zhang-Suen thinning algorithm (Zhang and Suen, 1984) to extract indoor topological networks from grid maps, providing an enriched framework for navigation.

Simultaneous Localization and Mapping (SLAM) has evolved to address the complex demands of construction sites. Visual-only SLAM, using monocular or stereo cameras, offers a lightweight and cost-effective solution but struggles with scale ambiguity and drift over time, limiting its long-term reliability (Macario Barros et al., 2022). VSLAM can be enhanced with using Inertial Measurement Units (IMUs) to improve robustness in dynamic environments (VI-SLAM) and by using depth sensors to facilitate dense 3D mapping (RGB-D SLAM) but they increase computational demands and increase the range constraints (Macario Barros et al., 2022). Traditional SLAM algorithms still struggle with occlusions and varying lighting conditions common on construction sites (Abaspur Kazerouni et al., 2022).

To overcome these limitations, recent research has integrated deep learning and semantic segmentation into SLAM pipelines, enhancing object recognition, scene understanding, and dynamic obstacle management, which may prove to be critical for construction sites (Pu et al., 2023). In any cases, SLAM strategies tailored for construction robotics must fuse various sensor modalities to improve accuracy in cluttered and dynamic workspaces (Yarovoi and Cho, 2024). Despite these advancements, ensuring real-time performance on resource-constrained platforms and reliable, non drifting, localization, are still challenging.

BIM-aided navigation extends these concepts by offering high-level representations of building structures that include geometric and semantic details, enabling robots to interpret and navigate environments with greater autonomy. Most BIM-driven studies utilize this data for pathfinding algorithms like Dijkstra (Wang et al., 2011), A* (Konakalla, 2014), and Fast Marching Method (FMM) (Garrido et al., 2013). In 3D contexts, Chen Q. et al., 2022 developed *BI3DM*, a voxel-based system mapping BIM information to enable navigation for drones and pedestrians. This approach integrates semantic and physical constraints to optimize paths, demonstrating its effectiveness across various BIM models. However, many of these approaches rely on manually pre-processed BIM data, which limits their responsiveness to the dynamic conditions typical of construction sites (Liu et al., 2021). Automating BIM data integration remains a critical challenge for real-time adaptability.

BIM’s utility extends beyond static navigation models, offering rich semantic data for dynamic and task-driven navigation. Studies like those by Gopee et al. (2023) converted BIM data into navigation maps, integrating semantic insights to guide stop-and-go operations and obstacle avoidance. Pauwels et al. (2023) investigated real-time semantic data transfer techniques, employing JavaScript Object Notation (JSON) and graph-based formats to construct semantic and grid maps for robotic workflows. Similarly, Zhou et al. (2020) proposed methods to extract indoor topological networks from BIM-based grids, creating integrated models for localization and navigation. Hendrikx et al. (2021) convert semantic entities present on BIM into a database of features that can be compared to the robot’s sensors and aid in localization. In 3D, Yin et al. (2023) translated BIM components into semantically enriched point cloud maps categorized by type and location. Their method employs a coarse-to-fine localization strategy, aligning LiDAR readings with semantic maps via iterative closest point (ICP) registration, enabling robust pose tracking with minimal sensor setups.

These efforts underscore BIM’s transformative potential in robotic navigation pipelines, improving localization, adaptability, and task planning in dynamic construction environments. However, gaps persist in integrating semantic data into comprehensive, accessible solutions. Many studies lack implementations that adhere to industry standards while offering intuitive, user-friendly systems that connect BIM at both global and local navigation levels. This paper aims to bridge these gaps by presenting an integrated, practical solution for BIM-aided navigation and task execution, demonstrating its utility in real-world construction scenarios.

### 2.2 User-centric design in construction automation

User-centric design is a critical area of research across all domains where robotic systems are gaining traction for commercial and industrial applications. This emphasis on usability is essential for achieving worker acceptance and operational efficiency (Jacob et al., 2023; Wewerka et al., 2020). In construction, the design and implementation of robotic systems must consider not only technical performance but also the social and psychological factors influencing human-robot interaction, including trust, perceived safety, and ease of use (Belzile et al., 2021).

In the broader field of human-robot interaction, efforts to create intuitive systems often focus on advanced interaction modalities such as gesture recognition, virtual reality interfaces, or natural language commands (Chen H. et al., 2022). However, these approaches may be impractical in industrial environments like construction sites, where robustness and simplicity are paramount (Braga et al., 2024). Many studies instead explore interfaces that simplify robot programming tasks, such as visual programming languages (Tilley and Gray, 2017). A notable concept involves programming robots through semantic representations of their capabilities in specific contexts (Steinmetz et al., 2018). While promising, this approach has not yet been applied in construction settings, particularly with map-based information systems that are critical for navigation and task execution.

The construction industry, characterized by dynamic and hazardous environments, presents unique challenges for automation (Yarovoi and Cho, 2024). In such settings, safety must take precedence in human-robot collaboration (HRC). Research indicates that workers are more likely to accept robots when their behavior is predictable, reliable, and seamlessly integrated into existing workflows. One effective approach is the use of dynamically generated safety zones around robots (Jacob et al., 2023; Braga et al., 2024). These zones mitigate risks such as collisions and distractions by adapting in real time to specific work scenarios, enhancing both trust and system flexibility (Walzer et al., 2022). Teleoperated systems, where human oversight plays a central role, are particularly favored in construction due to their ability to ensure safety and adaptability in high-risk scenarios.

Real-world deployments further underscore the importance of user-centric interfaces in fostering trust and achieving operational success (Walzer et al., 2022). By simplifying interactions, aligning robotic capabilities with user needs, and prioritizing safety, user-centric design principles enable smoother integration of robotics into construction workflows. As the industry increasingly adopts automation, embedding these principles into the development and deployment of robotic systems is vital for maximizing their potential.

### 2.3 Equipment detection and localization

Accurate localization of assets is crucial for managing materials, equipment, and tools on construction sites, which are often dynamic and challenging environments. Various technologies have been explored (Marcy et al., 2023), with some achieving commercial deployment, each offering distinct benefits and limitations.

Quick Response (QR) codes are a widely used and cost-effective solution for tagging materials and equipment. Their integration with BIM systems allows for quick access to asset data via mobile devices, improving communication and safety. However, QR codes require line-of-sight scanning and are prone to physical damage, which demands manual effort and makes them impractical for large-scale or fast-paced operations.

Radio-Frequency Identification (RFID) systems address many of these limitations by enabling contactless tracking without the need for direct visibility. Using electromagnetic signals, RFID tags and readers efficiently monitor materials and track progress (Omar and Nehdi, 2016), particularly when combined with BIM systems. However, environmental factors such as liquids or metals can interfere with signals, and deploying RFID infrastructure requires specialized equipment and careful calibration, adding complexity and cost (Xie et al., 2011).

UWB technology offers greater precision and reliability, even in obstructed environments. By leveraging trilateration (Yin et al., 2016), UWB enables real-time, accurate tracking of materials and equipment, making it particularly effective for detecting MEP assets. Despite these advantages, most UWB infrastructures require the installation of several fixed anchors in the field to compute trilateration, whose high cost restricts adoption.

Bluetooth Low Energy (BLE) provides a more affordable alternative, using signal strength to estimate asset locations (Marcy et al., 2023). Its compatibility with consumer-grade devices makes it flexible for smaller projects, but signal variability in complex environments limits its accuracy. As a result, BLE is better suited for general tracking tasks rather than high-precision localization.

Recent advances in artificial intelligence (AI) have introduced object detection systems using vision or LiDAR data. These solutions employ deep learning to identify and track assets based on visual or spatial features (Fernandes et al., 2021), offering rich contextual information and adaptability to dynamic environments. However, their reliance on computationally intensive models and high-quality sensors constrains their feasibility for real-time deployment in cost-sensitive projects.

Each of these technologies—QR codes, RFID, UWB, BLE, and AI object detectors—has unique strengths and trade-offs. By leveraging UWB in combination with BIM and robot localization, this work proposes a low-cost, scalable, and practical approach with minimal infrastructure requirements - no fixed anchors -addressing gaps in existing solutions and offering robust performance in dynamic construction environments.

## 3 Materials and methods

### 3.1 Semantic navigation strategy

This section outlines our approach, focusing on how information extracted from BIM is integrated into a two-level path planning system and a user-friendly semantic GUI.

#### 3.1.1 Topological semantic maps

**FIGURE 1:**
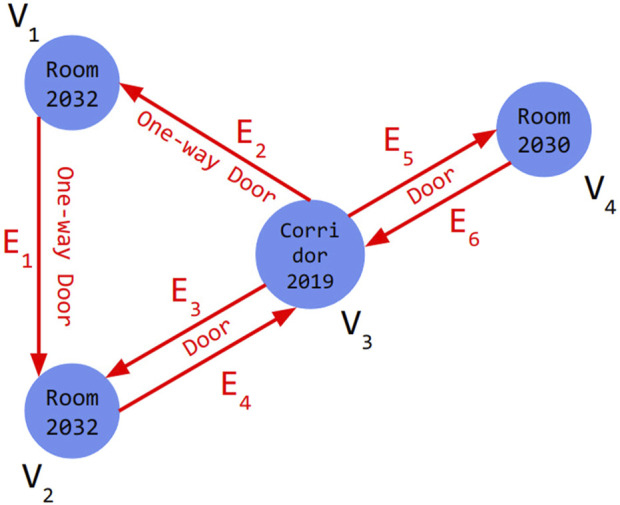
A directed hypergraph of S=(V,E) where $V=\left\{V_{1},V_{2},\ldots ,V_{n}\right\}$ is a set of nodes and $E=\left\{E_{1},E_{2},\ldots ,E_{m}\right\}$ is a set of hyperedges. Each node $\left(V_{i}\right)$ is an *IfcSpace* containing its relationships and each hyperedge $\left(E_{j}\right)$ is an *IfcDoor* with its attributes extracted by BIRS (Karimi et al., 2021).

Topological maps, enhanced with BIM-derived information, are instrumental for robot navigation. These maps structure building data into a graph of rooms, doors, and corridors enriched with geometric and semantic details (Strug and Ślusarczyk, 2017). Since BIM data encompasses vast information, identifying and translating relevant content for robotic systems is essential (Karimi et al., 2021). Such information is often modeled with Industry Foundation Classes (IFC), an open standard data schema for building and construction industry data, using classes such as *IfcSpace* and *IfcDoor* to describe the respective architectural elements. Here, we extend the directed hypergraph approach of (Palacz et al., 2019) by incorporating IFC semantics and geometry into a topological representation (Figure 1).

Nodes in the graph represent rooms, corridors, and other areas, and contain attributes such as room name, unique ID, center coordinates, area, wall materials, scan age, and potential hazards for the robot. Edges represent doors with attributes like unique ID, location, and opening direction. The directed hypergraph allows for multiple connections between nodes, so one-way paths are represented by a single directed edge, while paths without this restriction use two edges in both directions. Weights representing navigation costs are assigned to nodes and edges to guide navigation. The total weight of a node Vi is computed by Equation 1:

$$W_{V_{i}}=w_{m_{i}}+w_{a_{i}}+w_{s_{i}}+w_{h_{i}}$$ (1)

where $w_{m_{i}}$ depends on the walls material, $w_{a_{i}}$ , on the room area, $w_{s_{i}}$ , on the room scan-age, and $w_{s_{i}}$ , of the hazards in room i . Edge weights depend on door opening direction (e.g., for pushing, for pulling). The total path weight is computed by adding the total weights of all nodes and all edges contained in the path, as shown by Equation 2:

$$W=\overset{n}{\underset{i=1}{\sum }}W_{V_{i}}+\overset{m}{\underset{j=1}{\sum }}W_{E_{j}}$$ (2)

Paths with lower weights are prioritized. This system helps the robot avoid potential risks, such as sensor issues with glass walls, and ensures efficient navigation. Directed hypergraphs, incorporating *IfcDoor* attributes like coordinates and opening directions, facilitate assigning navigation costs. A Dynamo Script extracts IFC classes and parameters, storing data in an Extensible Markup Language (XML) database. A Python script translates this information into a ROS-compatible format, enabling seamless integration with the robot’s navigation system.

#### 3.1.2 Room-level semantic path planning

**FIGURE 2:**
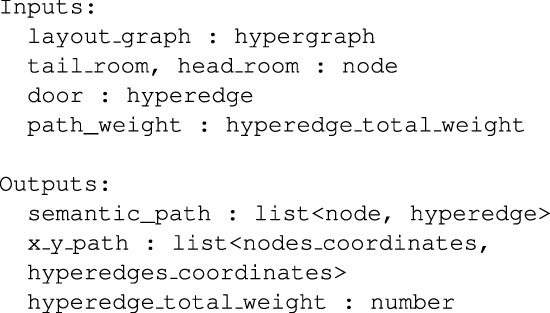
Data structure for IFC-based semantic optimal path planner algorithm.

The data structure of our semantic path planning algorithm is shown in Figure 2. Using the generated hypergraph, the start and end nodes are defined by the user. Our implementation employs a directed BF-hypergraph (Gallo et al., 1993), accommodating connections between multiple rooms. Forward and backward sub-hypergraphs represent bidirectional connections, incorporating door opening costs. The “Shortest Sum B-Tree” algorithm from Gallo et al. (1993) identifies optimal paths by minimizing cumulative weights. The output includes room and door coordinates in sequential order, guiding the robot’s navigation while providing semantic context for the user.

#### 3.1.3 Semantic graphical user interface design

The GUI, developed in Python notebooks, provides a semantic interface for intuitive robot operation. It integrates real-time data from ROS and BIM, displaying the building’s semantic information alongside live robot status. Users can:

• Select a destination from a drop-down list of rooms.

• View a live map showing the robot’s position and planned path.

• Adjust path planner weights (e.g., hazard, scan age) through an interactive panel.

• Trigger the robot’s movement by clicking the “Move Robot” button.

**FIGURE 3:**
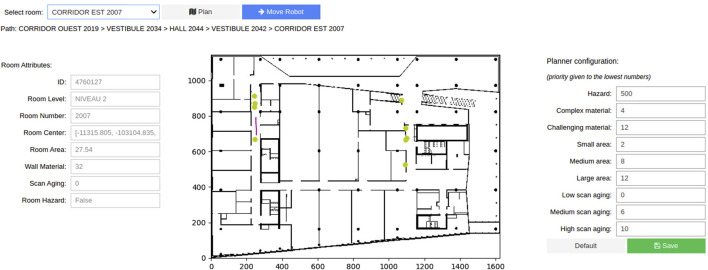
Semantic Graphical User Interface for the intuitive operation of a mobile robot with domain knowledge. The controls in the header allow selecting a destination and generating the path. The panel to the left shows the attributes of the selected room. The center contains a map of the environment, with the robot’s pose in real time being represented by the purple arrow. The center points of the rooms and doors in the path are represented in the map by the yellow circles. The right panel allows the user to reconfigure the different weights that are applied to the path generation.

This design bridges domain knowledge and robotic operations, enabling non-expert users to configure and monitor navigation tasks effectively (Figure 3).

#### 3.1.4 BIM-aware localization

**FIGURE 4:**
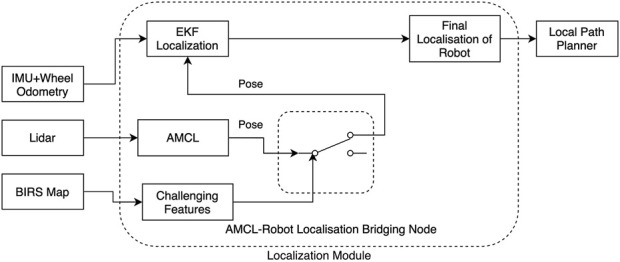
System overview of AMCL-Robot localization bridge which detects the presence of reliable AMCL pose and uses it to improve localization.

Low-level path planning, which operates within the framework of high-level plans, demands robust localization to adapt to the dynamic and often unpredictable nature of construction environments. Traditional localization methods, such as Adaptive Monte Carlo Localization (AMCL), face challenges in these settings due to environmental factors like dust, debris, and reflective surfaces, which can degrade accuracy. To address these limitations, we developed a BIM-aware localization technique that integrates AMCL (Thrun, 2002) with an extended Kalman filter (EKF) via a bridging node (Figure 4).

Before deployment, wall geometries extracted from the BIM model are used to generate an occupancy grid map of the building. This map is augmented with semantic information as described in Section 3.1.1, presented as a separate element list, and utilized by the localization module. During navigation, AMCL combines the occupancy grid map with odometry and laser scan data to provide real-time pose estimates for the robot. Simultaneously, the bridging node monitors the semantic elements associated with the robot’s current position on the map. In areas where certain sensing modalities are unreliable—such as near reflective surfaces like glass walls—the bridging node enhances accuracy by connecting AMCL outputs to EKF localization. This dual-layered approach leverages the detailed spatial and semantic data provided by BIM to correct discrepancies in AMCL-derived poses. A key feature of this strategy is its ability to mitigate the “pose jump” issue common with map-based localization methods like AMCL, where the robot might misidentify its position on the map.

When such errors are detected, the system temporarily switches to odometry-only dead-reckoning to navigate past the problematic zone. BIM data is critical in supporting this fallback strategy, as it provides precise spatial information to ensure the map-based localization is disabled for the minimum required duration. Extended reliance on dead-reckoning, as is well-known, introduces drift errors, emphasizing the importance of a tightly managed transition back to map-based localization once the challenging area is cleared.

### 3.2 UWB-based robot-centric equipment localization

As the robot navigates and updates its map, it simultaneously detects and localizes equipment on-site using active markers. Our approach employs ranging measurements between the robot and UWB beacons attached to the equipment. UWB, a radio technology operating at high frequencies (3.1–10.6 GHz), was chosen primarily for its high accuracy and robustness to occlusions. According to Jiménez and Seco (2017), UWB can achieve a precision of up to 10 cm, significantly outperforming alternatives such as BLE, which is often limited to a 5-m accuracy.

In this system, the task involves determining the locations of fixed UWB beacons based on the known positions of the robot. To accomplish this, we adapted the trilateration localization algorithm, commonly used to locate moving devices such as robots or humans, and inverted its application to localize static anchors instead (Yin et al., 2016).

As the robot navigates, its estimated position and the measured ranges to the UWB beacons are recorded at a synchronized frequency of 10 Hz. To simplify the system and improve accuracy while maintaining operational flexibility, the anchors are deployed at predefined, fixed heights. To enhance robustness against noise, ranging measurements are collected from 70 distinct robot positions distributed around each beacon. This aggregation of measurements results in an overdetermined system of equations, as shown in Equation 3:

$$\left\{\begin{array}{c} {\left(x_{1}-x\right)}^{2}+{\left(y_{1}-y\right)}^{2}+{\left(z_{1}-z_{a}\right)}^{2}=r_{1}^{2}\;\\ {\left(x_{2}-x\right)}^{2}+{\left(y_{2}-y\right)}^{2}+{\left(z_{2}-z_{a}\right)}^{2}=r_{2}^{2}\;\\ \ldots \;\\ {\left(x_{n}-x\right)}^{2}+{\left(y_{n}-y\right)}^{2}+{\left(z_{n}-z_{a}\right)}^{2}=r_{n}^{2}\; \end{array}\right.$$ (3)

where n=70 is the number of distinct ranging measurements being taken at different times; $r_{i}$ is the range measured at time t=i ; $x_{i}$ , $y_{i}$ , and $z_{i}$ are the known coordinates of the robot at time t=i ; $z_{a}$ is the known height of the anchor; and x , and y are the unknown coordinates of the anchor. An approximate solution for this system can be found using the Moore-Penrose generalized inverse method (Equations 4–6):

$$x={\left(A^{T}A\right)}^{-1}A^{T}b$$ (4)

with

$$A=\left[\begin{array}{cc} 2\left(x_{n}-x_{1}\right) & 2\left(y_{n}-y_{1}\right)\\ 2\left(x_{n}-x_{2}\right) & 2\left(y_{n}-y_{2}\right)\\ \ldots & \ldots \\ 2\left(x_{n}-x_{n-1}\right) & 2\left(y_{n}-y_{n-1}\right) \end{array}\right]$$ (5)

and

$$b=\left[\begin{array}{c} r_{1}^{2}-r_{n}^{2}-x_{1}^{2}-y_{1}^{2}-z_{1}^{2}+x_{n}^{2}+y_{n}^{2}+z_{n}^{2}-2z_{a}\left(z_{n}-z_{1}\right)\\ r_{2}^{2}-r_{n}^{2}-x_{2}^{2}-y_{2}^{2}-z_{2}^{2}+x_{n}^{2}+y_{n}^{2}+z_{n}^{2}-2z_{a}\left(z_{n}-z_{2}\right)\\ \ldots \\ r_{n-1}^{2}-r_{n}^{2}-x_{n-1}^{2}-y_{n-1}^{2}-z_{n-1}^{2}+x_{n}^{2}+y_{n}^{2}+z_{n}^{2}-2z_{a}\left(z_{n}-z_{n-1}\right) \end{array}\right]$$ (6)

The trilateration results provide an initial estimate, which is subsequently refined using the *Trust Region Reflective* algorithm, as shown in Equation 7 (Wang et al., 2017):

$$error=\overset{n}{\underset{i=1}{\sum }}|r_{i}-\left(\left\|x-{robot}_{i}\right\|\right)|$$ (7)

with $r_{i}$ : the range measured by the UWB device at t = i, **x**: the solution tested by the minimization algorithm, ${robot}_{i}$ : the robot position at t = i. This optimization technique minimizes a cost function that quantifies the discrepancy between the measured ranges and the estimated distances derived from the solution. The cost function is expressed as the sum of squared differences between the observed UWB ranges and the calculated distances based on the current beacon position estimate. This iterative refinement ensures a highly accurate localization of the beacons, even in noisy environments.

### 3.3 Experiments

We evaluated our approach through three sets of experiments: a simulation to validate the navigation system under challenging conditions, an experimental deployment using a real wheeled robotic platform, and a user study to assess the usability of the semantic GUI tool. These experiments involved navigating a building’s corridors while recording sensor data. We selected a building at École de technologie supérieure (Montréal, Canada), which, besides having a complete BIM model available, features multiple rooms, corridors, open spaces, and alternate paths to achieve the same navigation goal. This complexity allowed us to evaluate multiple features of the semantic path planner, such as dealing with blocked pathways and generating paths through unvisited places to maximize the explored area. Notably, this building also features a long corridor along a glass wall, which provided an ideal scenario to test the effect of the AMCL-Robot Localization bridging node on the localization performance. The robot was operated through the semantic GUI on a laptop, with high-level destination commands transmitted via a WiFi link. The robot’s global path planner processed these commands to generate waypoints, which were subsequently utilized by the local path planner to control the motors.

Running both simulated and real experiments served complementary purposes in evaluating our system. The simulation environment allowed us to test the navigation system under controlled conditions, ensuring that specific factors—such as BIM data integration, planned versus adjusted paths, and the semantic GUI—could be analyzed without external disruptions. Besides that, the user study was fully run on simulation to ease the study constraints on recruitment. The real-world experiments were essential to validate the feasibility of our approach in a practical, realistic scenario. These tests demonstrated how the system performs under real sensor noise, localization inaccuracies, and unexpected obstacles—factors that cannot always be fully replicated in simulation.

#### 3.3.1 Robotic platform and deployment

**FIGURE 5:**
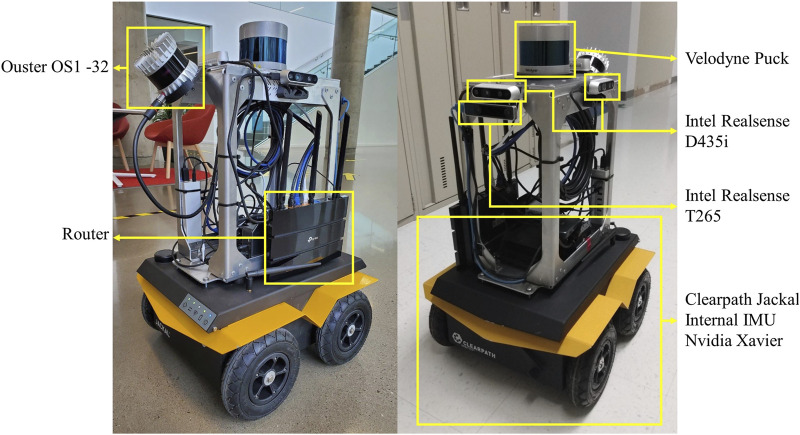
Mobile robot platform equipped with various sensors.

The robotic platform used for this study was a Clearpath Jackal, a four-wheeled unmanned ground vehicle equipped with a hybrid vision and laser sensing system, as shown in Figure 5. With our sensors and software stack, the Jackal is capable of semi-autonomous navigation, with a remote operator issuing high-level commands.

For odometry estimation, the base platform streams wheel encoders and an Inertial Measurement Unit (IMU) data to an NVidia Jetson onboard computer that processes them and executes navigation algorithms. Clearpath’s suite of ROS nodes for control, state estimation, and diagnostics provided the foundation for robot operation. The sensing system is designed for digital twin data collection and includes LiDARs and depth cameras to comprehensively monitor the robot’s surroundings. A front-facing Intel Realsense T265 tracking camera combines visual data with wheel odometry to provide accurate localization. The Intel Realsense D435i depth camera feeds depth images to a collision avoidance module, enabling the robot to react when nearing obstacles. A horizontally mounted Velodyne Puck 32MR LiDAR scans the walls for global localization inside the known map. Additional sensors, including three more D435i cameras and an Ouster LiDAR, are integrated for richer data collection. For assets detection, the robot is equipped with a UWB Pozyx tag placed 78 cm above the floor level (not visible on the picture).

**FIGURE 6:**
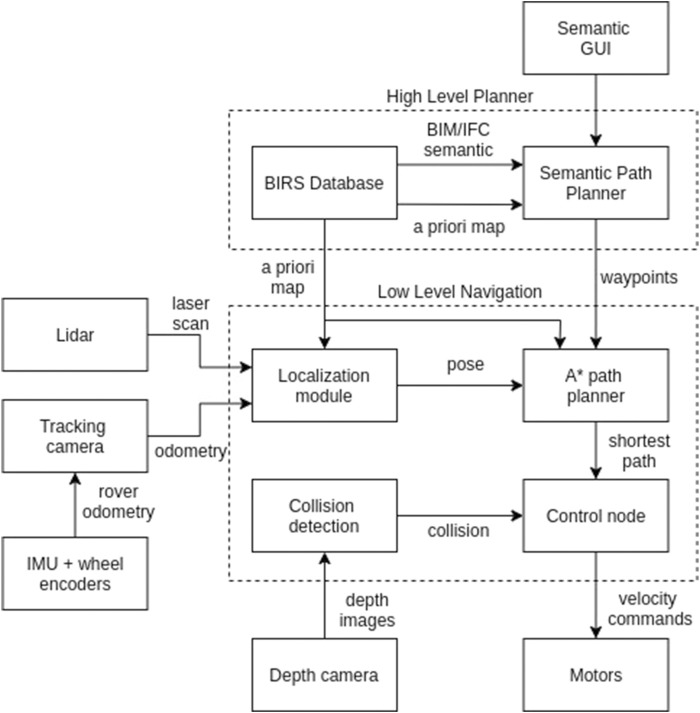
System Overview: A high level planner that process BIM/IFC information and user inputs is integrated to a low level navigation stack in a cascade design. The low-level module takes care of the localization, local path planning and collision avoidance tasks, while the high-level planner generates paths based on BIM/IFC semantics.

Figure 6 illustrates the system architecture. The robot’s pose within the map is estimated using the ROS implementation of our localization strategy, integrating BIM semantic, described in Section 3.1.4. When a destination room is selected by the operator via the GUI, the semantic path planner outputs a preferred route going through the center points of rooms, doors, and corridors along the path. The local mapper (A* algorithm (Hart et al., 1968)) is then used to compute the shortest path from the robot’s current position to the next waypoint. Velocity commands derived from the path are sent to the robot’s internal controller to execute navigation through the planned route while reacting to obstacles detected by the depth camera.

#### 3.3.2 Simulation

**FIGURE 7:**
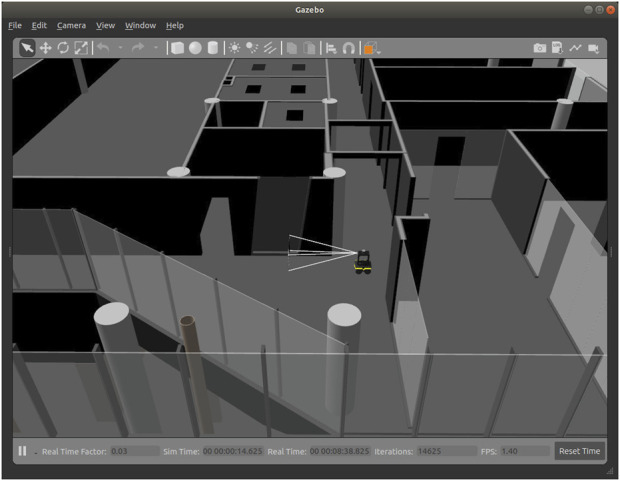
View of the simulated environment used to test the BIM/IFC optimal path planning approach. The building 3D model was built with geometry information extracted from the BIM. The robot model simulates the sensors and possesses the same characteristics as the real robot.

The simulation experiments were conducted using the Gazebo Simulator, leveraging its capability to create accurate digital twins of real-world environments. Building information from the BIM model was exported to generate a detailed 3D representation of the environment. This digital twin provided a realistic testbed for evaluating the robot’s navigation performance. Figure 7 illustrates the simulated robot navigating its environment, complete with varying wall textures and transparency to replicate the diverse visual conditions encountered during deployment.

#### 3.3.3 User study design

To evaluate the usability, efficiency, and safety of our semantic navigation system compared to conventional methods, we conducted a user study with 22 participants. They controlled a robot in simulation using two distinct modes: one leveraging our semantic navigation algorithm and another employing a conventional approach. The task involved exploring a simulated building, collecting data at specified locations, and avoiding a designated hazardous area. We recorded performance metrics, including task completion time, area explored, and number of actions performed. Participants also completed a usability evaluation questionnaire, including the NASA Task Load Index (NASA-TLX), to assess workload and user satisfaction.

The 22 participants in the user study were members of our research group, representing diverse backgrounds, including robotics (59%), construction engineering (18%), and others (22%) such as mechanical engineering, biology, and psychology. The inclusion of individuals not experts in robotics allowed us to assess the system’s usability across varying levels of technical expertise. We discuss potential impacts of the distribution of participants’ backgrounds in the Results section.

**FIGURE 8:**
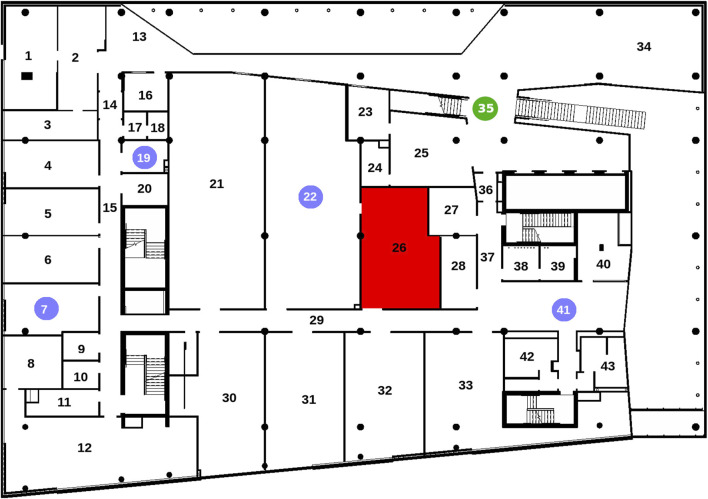
Map explored by the participants during the user study. The green circle indicates the starting position of the robot, the blue circles are rooms that should be visited and the red area should be avoided.

The experimental environment is depicted in Figure 8. The robot started at the green circle, and participants were tasked with visiting the four rooms marked with blue circles, returning to the starting point afterward. The red-marked room represented a construction zone and was off-limits. Each participant completed this exploration task twice: once using the semantic navigation mode and once with the conventional control mode, with the order randomized. A time limit of 15 min was imposed for each task to simulate real-world constraints where users must complete tasks under time pressure and to prevent participants from engaging in non-task-related exploration, ensuring that collected data accurately reflected system performance. Participants had access to a reference document summarizing the objectives and listing the names of all rooms.

The custom GUI was the only interface for participants to interact with the simulation. The GUI displayed the robot’s current pose, the navigation path, and interactive controls tailored to the specific control mode:

• Basic control mode: Participants clicked on any point on the map to set a navigation goal. Pressing the “Plan” button triggered an A* algorithm to calculate the shortest path from the robot’s current position to the selected point, which was displayed on the map. Additional “Move” and “Stop” buttons allowed participants to control the robot’s movement.

• Semantic control mode: Participants selected a room from a dropdown list of names, enabling the semantic navigation algorithm to generate a path to the chosen room. Like the conventional mode, “Move” and “Stop” buttons controlled the robot’s actions.

## 4 Results

### 4.1 Localizing assets in real-world settings

**FIGURE 9:**
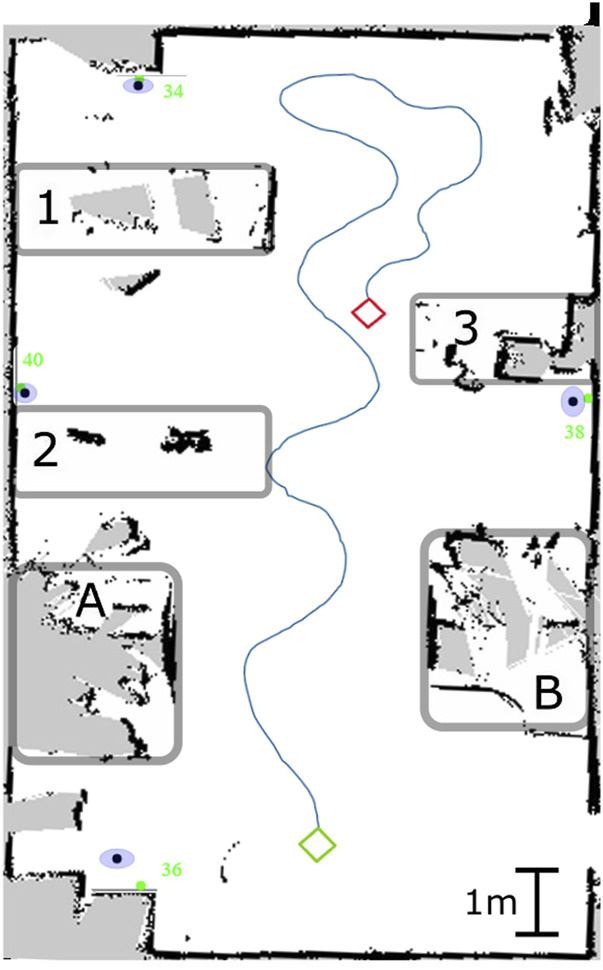
Robot’s winding trajectory in the test room: the green diamond represents the initial position and the red one represents the final position. The anchors are indicated with the green dots. The mean detected positions are the black dots and the blue ellipses represent the standard deviation of the measurements. A and B are 1.68 m high obstacles and 1 to 3 are 0.70 cm high ones.

To evaluate the active equipment localization algorithm, we performed a series of tests with the Jackal robot and four UWB beacons representing equipment, positioned in a room according to Figure 9. The Figure shows the robot mapping of the test room including line-of-sight obstructions: the boxes A and B are 1.65 m high and the boxes 1 to 3 are 70 cm high.

As discussed previously, the positions considered for the 2D trilateration have to be non-colinear, otherwise the algorithm will output erroneous results. For that reason, the robot’s trajectory must be winding in order to ensure it. A 10 cm interval between consecutive positions is also enforced in the algorithm. Figure 9 shows an example of a winding trajectory on a generated plan of the test room.

After cumulating 20 detections per anchor, we obtained the results presented in Table 1, where D is the distance from the origin. The results show that the mean error on the detection is around 11 cm on both x and y axes. The worst results come from the tag *36* which is partially occluded. The minimal error is about 1 cm while the maximal goes up to 63 cm. Figure 9 also presents a graphical representation of the error and standard deviation for each tag. The green dots represent the ground truth, the black dots are the mean position detected by the algorithm and the blue ellipses represent the standard deviation of the detections.

**TABLE 1:** UWB-beacons detection errors in meters.

Error (m)	Mean	Min.	Max.
X	0.119	0.0116	0.489
Y	0.115	0.0119	0.638
D	0.0684	0.000105	0.294

### 4.2 Navigation performance

We then navigate the robotic system out of the test room to validate it in a series of tests with three primary objectives::

1. Assess the accuracy of the AMCL-Robot Localization bridge when navigating challenging environments, such as areas with glass walls.
2. Evaluate the effectiveness of the semantic path planner in generating optimal paths using building information from BIM/IFC.
3. Examine how modifications to the building information influence the paths generated by the system.

**FIGURE 10:**
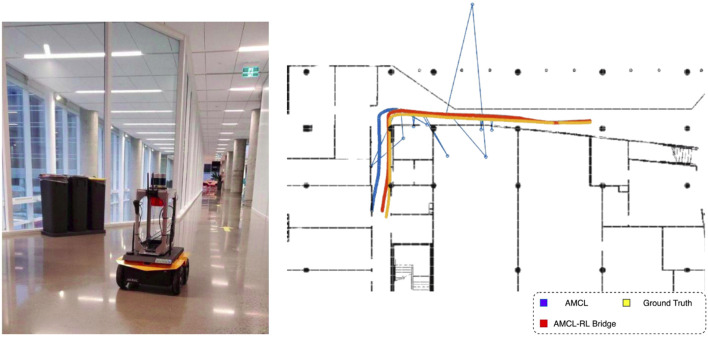
The AMCL bridging node improves localization accuracy near glass walls. (**left**) Robot moving near a large glass wall. (**right**) Performance of AMCL bridging node compared to the AMCL localization in that section.

To test the accuracy of the AMCL-Robot Localization bridge, we selected a corridor with a glass wall—a challenging scenario for conventional localization strategies. AMCL relies heavily on LiDAR distance measurements, which can be inconsistent when encountering glass surfaces, sometimes detecting the glass as an obstacle and other times ignoring it. Figure 10 illustrates a comparison between standard localization and the enhanced AMCL-Robot Localization bridge under such conditions. When the BIM-aware switching mechanism, described in Section 3.1.4, is triggered by the semantic information of an upcoming challenging feature, at the corner before the glass wall, it significantly improves localization accuracy. For subsequent tests, only the best localization method was used.

**FIGURE 11:**
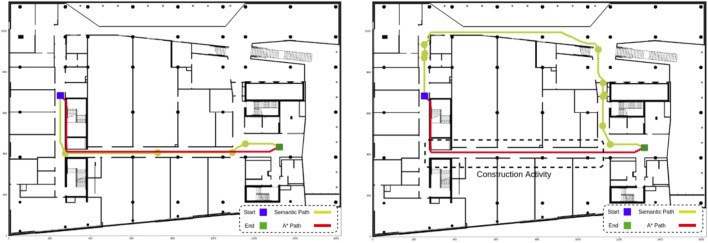
High-level and low-level paths: A* generates the shortest path possible between start and end, not taking advantage of the BIM/IFC semantics. (**left**) Without any special condition, both algorithms generate the shortest path. (**right**) When a construction activity is happening, our semantic path planner is able to find an alternative path, while A* causes the robot to navigate through the hazardous area.

The semantic global path planner was evaluated in a scenario where the robot started in a western corridor (CORRIDOR OUEST) and needed to reach an eastern open area (CORRIDOR EST). Figure 11’s left-hand side displays the building map and the path generated by a standard A* algorithm, marked in red. This path represents the shortest route between the two points, considering only the building geometry and a minimal safety margin around the robot. When the semantic global path planner was applied to the same scenario, a comparable path (marked in yellow) was generated, as there were no hazards, doors, or undesirable materials on the route. The algorithm identified and listed the rooms along the path, allowing the user to intuitively monitor the robot’s progress through the GUI, as shown in Figure 3. Additionally, waypoints representing the centers of rooms and doors were provided, enabling safer and more precise navigation, especially in challenging environments like glass doors that are less detectable by sensors. The semantic information aids the A* algorithm in finding the shortest and safest path between waypoints.

In a subsequent test, the building information was updated to simulate a construction activity in a specific area, marked by a dashed box in Figure 11’s right-hand side. The semantic global path planner automatically adjusted the route to avoid the hazardous zone, generating an alternative path (yellow). The system flagged the hazard through the GUI, allowing the operator to decide whether to proceed with scanning the area or postpone the task. The alternative path prioritized safety over distance, demonstrating the planner’s adaptability to dynamic environments. This scenario also provided an opportunity to test the AMCL-Robot Localization bridge in proximity to a large curtain wall, where the bridge enhanced the robot’s positional accuracy by integrating BIM-derived spatial information with sensor data. The semantic GUI further enhances usability by displaying the aging of scanned rooms, enabling operators to select destinations for data collection strategically. This feature supports efficient robot deployment, allowing operators to plan multiple missions with minimal redundancy and improved coverage.

**FIGURE 12:**
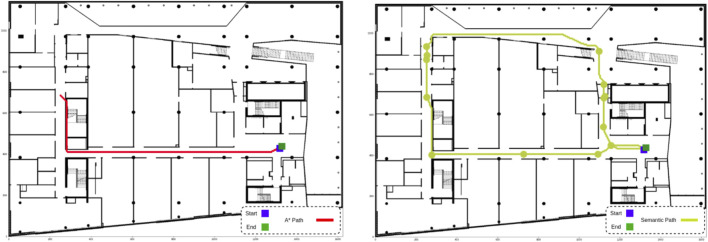
BIM/IFC semantics can be used to increase explored area. (**left**) By searching for the shortest possible path, A* causes the robot to navigate the same corridor twice. (**right**) The semantic path planner avoids recently explored areas, resulting in paths that cover more of the environment.

Finally, the robot was tasked with navigating to a destination and then returning to the starting point. Figure 12’s left-hand side shows the path generated by the A* algorithm, which retraced the same corridor on the return trip, minimizing the explored area. By contrast, the semantic path planner, leveraging BIM/IFC semantics, tracked the time since each room was last visited and prioritized unexplored zones for the return trip. Figure 12’s right hand side illustrates how this approach led to greater environmental coverage, enabling the robot to collect more comprehensive data. This cascade navigation system, integrating BIM-ROS information, proved effective for autonomous and precise data collection in construction environments.

### 4.3 User study

To evaluate the efficiency of each control mode from the operator’s perspective, we analyzed three metrics: the number of input actions (clicks), task completion time, and the size of the explored area. Efficiency was defined by minimizing operator input and reducing task duration. Since the semantic algorithm is designed to position the robot centrally within rooms and corridors, it was hypothesized to enable broader area coverage and improved data collection compared to the basic control mode.

A Wilcoxon signed-rank test with a 95% confidence interval was conducted to assess the statistical significance of observed differences between the two modes.

Table 2 shows the average clicks per minute for each mode. Participants using the basic control mode clicked, on average, 1.37 times more per minute than those using the semantic mode. This difference, confirmed by a p-value of $3.9\times 10^{-6}$ , reflects user behaviors during navigation: participants often explored multiple paths to the same destination, corrected trajectories, or set multiple intermediate waypoints. In contrast, the semantic algorithm efficiently calculated optimal paths, reducing the need for such adjustments. This difference and statistical significance is consistent among the subgroups of roboticists and non-experts.

**TABLE 2:** Average clicks per minute.


Basic mode	Map	Plan	Move	Stop	Total
	0.87	0.72	0.64	0.12	2.36
Semantic mode	Room select		Move	Stop	Total
	0.45		0.39	0.15	0.99

Table 3 details the differences in completion time and explored area, alongside their statistical significance. On average, the basic control mode required 47 additional seconds to complete tasks. This was expected, as participants in the basic mode spent more time evaluating routes, experimenting with pathways, and adjusting movements based on A* algorithm feedback. However, the t-test revealed that the difference in completion times was not statistically significant. Further analysis indicated that longer durations were typically associated with each participant’s first run, regardless of the mode, suggesting that extended times were due to participants familiarizing themselves with the environment rather than inherent inefficiencies of the basic mode.

**TABLE 3:** Average difference of completion time and explored area between the two control modes, and corresponding p-value.

	Difference	p-value
Time (sec)	−47	0.306
Area $\left(m^{2}\right)$	80.79	0.011

The semantic control mode significantly improved area coverage, with participants exploring an average of 80.79 square meters more than in the basic mode. This improvement stems from the semantic mode’s approach, which prioritizes navigation through the center points of rooms and corridors, optimizing coverage. Statistical analysis confirmed this effect, as indicated by the t-test results. However, when comparing participants with robotics expertise to those without, we observed that roboticists often achieved comparable coverage using the traditional path planning strategy. As a result, for this subgroup, the difference between the two modes was not statistically significant (p-value = 0.289).

Safety assessments focused on the frequency of paths crossing hazardous areas. In the basic mode, participants’ paths crossed the hazardous “red room” in 31.8% of runs. While most participants corrected their trajectories upon noticing the error, one instance remained uncorrected, which would pose a significant safety risk in a real-world scenario. In contrast, the semantic mode consistently avoided hazardous areas, leveraging BIM data to assign higher risk weights to such zones. This feature minimized human errors, demonstrating a key advantage of integrating semantic reasoning into navigation strategies.

Usability was evaluated through a structured survey based on the QUEAD questionnaire (Schmidtler et al., 2017) and the NASA-TLX workload assessment (Hart and Staveland, 1988). QUEAD, originally designed to assess the perceived usability and acceptance of assistive devices, was adapted for this study by including all relevant questions while omitting, for instance, those related to physical interactions. The full NASA-TLX questionnaire was applied, covering six dimensions of task load assessment. The final survey^1^ consisted of 21 questions, each rated on a seven-point Likert scale, measuring perceived ease of use, cognitive load, and user preference for both navigation modes.

**FIGURE 13:**
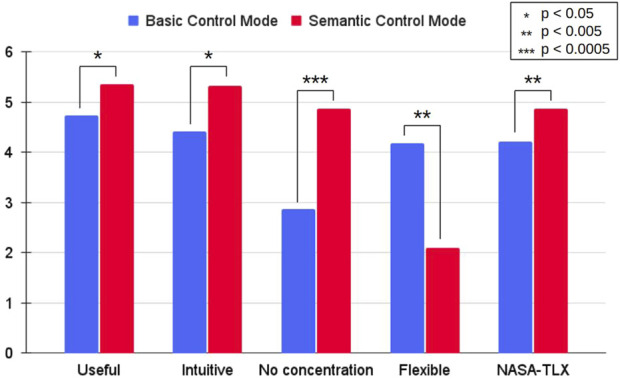
Average score attributed to four relevant questions and NASA-TLX score using a seven-point Likert scale (0–6). Higher values mean more positive perception by the participants and less demanding task load. All aspects were found to be statistically significant (p-value <0.05 ).

Figure 13 displays average responses to the four most relevant survey questions, alongside the average NASA-TLX composite score, which averages all six dimensions. Higher scores indicate greater ease of use or lower cognitive demand. Statistical analysis revealed that participants found the semantic mode more useful, intuitive, and less cognitively demanding (p-value <0.05 ). However, the basic mode was perceived as more flexible, appealing to participants who preferred greater control for tasks requiring precise navigation.

When analyzing survey responses separately for roboticists and non-roboticists, the same overall trends were observed across all categories. However, statistical analysis revealed p-values exceeding 0.05 for the Useful and Intuitive categories among roboticists, and for the Flexible category among non-roboticists. This suggests that roboticists, being more familiar with control systems, were equally comfortable with both navigation modes, whereas the semantic control mode had a stronger impact on usability perceptions among non-experts.

Across 44 unique runs, 10 instances were excluded from analysis due to issues such as the path planner failing to identify viable paths or the robot experiencing disorientation from rapid command inputs, which affected localization performance. These challenges highlight potential areas for refinement in both path planning and control strategies to improve system robustness.

Overall, the semantic control mode proved to be more efficient, requiring fewer user inputs, improving area coverage, and ensuring safer navigation by automatically avoiding hazardous areas without user intervention. However, 6 out of 22 participants expressed a preference for the basic control mode, citing its greater flexibility for precise navigation. This feedback suggests an opportunity to combine the strengths of both approaches. A hybrid control mode - where the semantic algorithm generates a baseline path, but users retain the ability to make fine adjustments—could balance automation’s efficiency with user-directed adaptability, making it more suitable for diverse operational needs.

## 5 Discussion

Beyond addressing the problem statement, our findings contribute to both theoretical advancements in robotic navigation and practical applications for real-world deployment. The integration of BIM with real-time sensing represents a significant step forward in robotic decision-making for highly dynamic construction sites, where traditional approaches struggle due to frequent and unpredictable changes. Through simulation and real-world deployments, we demonstrated the system’s capability to navigate complex layouts while maintaining localization accuracy and usability, which are critical for enabling autonomous robots in practical construction scenarios.

One of the key contributions of this work is the cascade navigation stack, which advances BIM-based navigation beyond static path planning. Existing BIM-driven navigation frameworks often rely on precomputed routes derived from digital building models, limiting their adaptability to unforeseen environmental changes, or propose navigation strategies coupling tightly BIM and path planing. In contrast, our approach dynamically integrates high-level semantic information from BIM with real-time local sensing and path planning, allowing for continuous path adjustments in response to evolving site conditions. This hybrid methodology bridges the gap between global planning and real-time perception, ensuring more reliable navigation in environments where temporary structures, moving equipment, and shifting obstacles are common. Both simulation and real-world experiments confirm that the system effectively adjusts its trajectory based on site dynamics, demonstrating the feasibility of BIM-enhanced adaptive navigation as a scalable and robust solution for construction robotics. Our validation was performed only on AMCL based on 2D lidar scans, but it could be combined with more recent AI-powered localization and SLAM approaches.

Another major contribution of this research is the semantic graphical user interface (GUI), which enhances accessibility for non-expert users. One of the challenges in deploying autonomous robots on construction sites is the reliance on specialized operators, which limits large-scale adoption. Our GUI simplifies robot operation through an intuitive, visually guided interface that leverages BIM data for navigation. The user study showed that participants from diverse backgrounds—ranging from robotics to construction engineering—could effectively interact with the system, achieving improved task efficiency while experiencing reduced cognitive load. This suggests that integrating semantic and interactive elements into robot control interfaces can bridge the gap between automation and construction practitioners, making robotic solutions more practical for real-world applications.

Despite these advancements, certain challenges remain regarding real-world deployment. Construction sites are highly dynamic and unstructured environments, and while our approach incorporates real-time sensing to update navigation paths, adapting BIM models to reflect constantly changing site conditions remains an area for improvement. Current BIM frameworks, while useful for *a priori* planning, are not always updated frequently enough to account for rapid field changes, such as newly placed scaffolding, temporary work zones, or shifting material stacks. Although our system partially mitigates this through real-time obstacle detection, future research could focus on automated BIM updates based on live sensor data, ensuring the digital model remains aligned with site conditions. Furthermore, while our system is designed for indoor construction environments, adapting it for outdoor or large-scale projects would require modifications, particularly in cases where BIM data is incomplete or unavailable. Integrating alternative spatial data sources, such as drone-based photogrammetry or real-time LiDAR mapping, could further expand its applicability to broader construction scenarios.

Beyond technical challenges, practical industry adoption is a critical factor. The construction sector has historically been slow to adopt automation technologies due to concerns over cost, reliability, and workforce adaptation. While our study demonstrates the feasibility of BIM-driven robotic navigation, further validation in real-world workflows is necessary to ensure seamless integration with existing site operations. Future research could involve collaboration with industry professionals to assess the system’s impact on productivity, safety, and efficiency over extended deployments. Additionally, optimizing the cost-effectiveness of UWB-based asset detection could enhance scalability, making it viable for large construction projects requiring precise tracking of multiple assets.

Furthermore, long-term usability and workforce adaptation remain crucial aspects of practical deployment. While the user study provided initial validation, further research is needed to understand how construction professionals interact with the system over extended periods. Studies involving a broader participant base that better represents the potential end-users in the construction industry are necessary to enhance the generalization and applicability of our findings. Longitudinal studies assessing the learning curve, long-term adoption, and potential modifications to enhance ease of use would provide valuable insights into ensuring effective integration into real-world construction workflows.

Looking ahead, this framework can be expanded to include markerless detection of Mechanical, Electrical, and Plumbing (MEP) elements, leveraging computer vision and AI-based object detection techniques. Enhancing the user interface with customizable options for dynamic site conditions will improve adaptability and usability. Refining the high-level path planner by incorporating normalized weight assignments for topological mapping will allow further customization based on application requirements. These enhancements will optimize semantic navigation and further integrate robotics into construction workflows.

The findings from this research contribute to ongoing discussions in autonomous construction robotics, BIM-driven navigation, and human-robot interaction. Future work should focus on enhancing semantic scene understanding by fusing BIM with real-time perception data, optimizing sensor fusion strategies for improved localization accuracy, and conducting extended usability studies with industry professionals. These efforts will further solidify the role of BIM-integrated robotics in automated construction, paving the way for more intelligent, adaptable, and user-friendly robotic solutions aligned with industry needs. By addressing both technological and usability barriers, this study provides a foundation for continued advancements in construction automation, demonstrating how robotics can be effectively integrated into complex and evolving work environments.

## 6 Conclusion

This paper presented a comprehensive framework for BIM-aided semantic navigation and asset localization, aimed at enhancing the efficiency and safety of autonomous robotic operations on construction sites. Leveraging the BIRS system, building information was extracted from IFC schemas and structured into hypergraphs for path planning. Weighted connections in the hypergraph encoded conditions affecting navigation, enabling the semantic path planner to prioritize safe and efficient routes. These high-level paths were seamlessly integrated with a low-level navigation system, where the A* algorithm calculated the shortest feasible paths within the optimal semantic route. Additionally, a BIM-aided localization strategy improved the robustness of the AMCL algorithm, particularly in challenging scenarios such as navigating near glass walls.

The framework’s effectiveness was validated through simulated and real-world case studies, demonstrating its adaptability to dynamic site conditions. Furthermore, the proposed active marker-based equipment detection strategy achieved a mean localization error of less than 12 cm, without requiring expensive infrastructure, showcasing its practicality for asset tracking in construction activities.

By addressing these advancements, the proposed system contributes to the future of safe, smart, and sustainable robotic operations in dynamic construction environments, offering a scalable solution that meets industry needs.

## Data Availability

The raw data supporting the conclusion of this article will be made available by the authors, without undue reservation.

## Footnotes

1 Survey available at https://forms.gle/19fwfJVr9sKJQZvZ8
